# Validity and Reliability of the Richmond Agitation-Sedation Scale in Pediatric Intensive Care Patients: A Multicenter Study

**DOI:** 10.3389/fped.2021.795487

**Published:** 2022-01-03

**Authors:** Rocío Tapia, Jesús López-Herce, Ángel Arias, Jimena del Castillo, Santiago Mencía

**Affiliations:** ^1^Pediatric Intensive Care Unit, Hospital Universitario Ramón y Cajal, Madrid, Spain; ^2^Instituto Ramón y Cajal de Investigación Sanitaria (“IRYCIS”), Madrid, Spain; ^3^Pediatric Intensive Care Unit, Hospital General Universitario Gregorio Marañón, Madrid, Spain; ^4^Maternal and Child Health Department (“Red SAMID”), Universidad Complutense, Madrid, Spain; ^5^Gregorio Marañón Health Research Institute, Madrid, Spain; ^6^Research Support Unit, Hospital General Mancha Centro, Alcázar de San Juan, Spain

**Keywords:** anesthesia and analgesia, intensive care unit, pediatric, monitoring, physiologic, nursing assessment, validation studies as topic, reproducibility of results

## Abstract

**Background:** There is limited data about the psychometric properties of the Richmond Agitation-Sedation Scale (RASS) in children. This study aims to analyze the validity and reliability of the RASS in assessing sedation and agitation in critically ill children.

**Methods:** A multicenter prospective study in children admitted to pediatric intensive care, aged between 1 month and 18 years. Twenty-eight observers from 14 PICUs (pediatric intensive care units) participated. Every observation was assessed by 4 observers: 2 nurses and 2 pediatric intensivists. We analyzed RASS inter-rater reliability, construct validity by comparing RASS to the COMFORT behavior (COMFORT-B) scale and the numeric rating scale (NRS), and by its ability to distinguish between levels of sedation, and responsiveness to changes in sedative dose levels.

**Results:** 139 episodes in 55 patients were analyzed, with a median age 3.6 years (interquartile range 0.7–7.8). Inter-rater reliability was excellent, weighted kappa (κ_w_) 0.946 (95% CI, 0.93–0.96; *p* < 0.001). RASS correlation with COMFORT-B scale, rho = 0.935 (*p* < 0.001) and NRS, rho = 0.958 (*p* < 0.001) was excellent. The RASS scores were significantly different (*p* < 0.001) for the 3 sedation categories (over-sedation, optimum and under-sedation) of the COMFORT-B scale, with a good agreement between both scales, κ_w_ 0.827 (95% CI, 0.789–0.865; *p* < 0.001), κ 0.762 (95% CI, 0.713–0.811, *p* < 0.001). A significant change in RASS scores (*p* < 0.001) was recorded with the variance of sedative doses.

**Conclusions:** The RASS showed good measurement properties in PICU, in terms of inter-rater reliability, construct validity, and responsiveness. These properties, including its ability to categorize the patients into deep sedation, moderate-light sedation, and agitation, makes the RASS a useful instrument for monitoring sedation in PICU.

## Introduction

International clinical guidelines recommend monitoring sedation in critically ill children with a validated and age-appropriate scale (1, 2). This allows to assess the depth of sedation in a standardized way and adjust objective-guided treatment, to better avoid over and under-sedation (1–3).

A gold standard reference scale does not exist (4, 5). The COMFORT scale, its modified version COMFORT behavior (COMFORT-B), and the State Behavioral Scale (SBS) are the most recommended in the international sedation guidelines (1, 2). The COMFORT-B scale is used the most (6–9). It has shown high reliability, construct validity and responsiveness in the pediatric intensive care unit (PICU), in ventilated and non-ventilated patients (5, 10–13). It distinguishes among 3 levels of sedation/agitation, for which the authors recommend associating it to a second scale, such as the Nurse Interpretation of Sedation Score (NISS) (3, 5). The greatest disadvantage of the COMFORT-B scale is the time required to perform it (14). The other recommended scale, the SBS, is only valid for ventilated patients (15). For these reasons, it would be useful to have a simpler alternative, capable of quickly differentiating between different levels of sedation, which would not require the use of other complementary scales, and apt to intubated and non-intubated patients.

The RASS (Richmond Agitation-Sedation Scale) is commonly used in adults admitted to critical care (16, 17). In critically ill children, RASS is used as a starting point for delirium diagnosis along with the Pediatric Confusion Assessment Method for the ICU (pCAM-ICU), Preschool Confusion Assessment Method for the ICU (psCAM-ICU) and Cornell scales, which require a RASS score ≥-3 to start assessment (18–20).

Although the RASS has not been sufficiently validated in critically ill children, some PICUs use it for sedation monitoring, due to the simplicity and quickness of the procedure (3, 21, 22). Only two previous studies have analyzed RASS inter-rater reliability in critically ill children, obtaining good results in this population (21, 22). However, these studies have their limitations, as they are single-center studies. RASS construct validity has also been explored in one of them, using the University of Michigan Sedation Scale (UMSS) as the comparator instrument, a validated sedation scale for pediatric procedures which does not include agitation (21). Agitation was only analyzed based on the expert opinion, using a visual analog sedation-agitation scale (VAS) (21).

The aim of our study was to analyze the measurement properties of the RASS in children admitted to PICU, in terms of inter-rater reliability, construct validity and responsiveness, in a prospective multi-center study.

## Materials and Methods

A prospective multi-center study was carried out, with the participation of 14 Spanish PICUs. The study was approved by the Research Ethics Committee of the promoting center. Written informed consent of parents and mature minors subjects was obtained.

### Study Period

Ethics committee approval in July 2016, video recording of the episodes from August to November 2016, theoretical training in June 2018, and video evaluation (training and final) from July 2018 to February 2019.

### Patients

Patients admitted to the PICU of the promoting hospital were enrolled until a minimum of 100 episodes from 50 patients was reached (following the COSMIN recommendations for an adequate sample size) (23). Patients between ages 1 month and 18 years with any level of sedation were included. Exclusion criteria were uncontrolled pain, severe psychomotor impairment, auditive or visual impairment, neuromuscular diseases, and treatment with muscle relaxants.

### Research Team

The research team consisted of 14 intensive pediatric doctors, including the principal investigator, and 14 PICU nurses. The pediatricians belonged to the Analgesia and Sedation Group of the Spanish Society of Pediatric Intensive Care (SECIP). The nursing staff had more than 10 years of experience in the PICU. Eighty-four percent of the researchers had previous experience using the COMFORT-B scale.

The research team received a training course in the application of the RASS and COMFORT-B scales, following the instructions published by their authors (24, 25). The training course consisted of an in-person theoretical-practical section of 2 h, and a second non-attendance part in which every researcher applied the scales in 20 video recorded clinical cases.

### Video Recordings of the Episodes

The principal investigator carried out the patients' video recordings according to the following protocol: (1st) observation of the patient without stimulation, including all parts of the body; (2nd) broadcast of auditory stimulus, calling the patient by their name, telling them to open their eyes and to look at the interlocutor; (3rd) muscle tone assessment, holding and dropping one arm; (4th) application of a tactile stimulus of increasing intensity, from a gentle touch to the shoulder to a potentially painful stimulus, following the RASS instructions. The ventilator screen and the vital signs monitor were also recorded. In order not to influence the observers, the stimulation sequence was done until the end, even if a response appeared in the first steps, except if the patient's agitation prevented it. The same patient could be analyzed once a day for several days or several times in the same day, if any change in sedation was made.

### Scales Assessment

The researchers were randomly divided into 7 groups of 4 members each (2 nurses and 2 pediatricians), equally dividing the total number of episodes to be analyzed among the 7 groups, so as not to overburden the collaborators. The same episode was independently assessed by the 4 researchers. Each researcher scored the RASS (Supplemental Appendix 1) first, the COMFORT-B scale (Supplemental Appendix 2) second, and the NRS third, of their corresponding episodes.

The RASS consists of 10 levels of sedation/agitation: 5 of sedation, one of calm alertness and 4 levels of agitation (24). Each value on the RASS scale is defined in 2 complementary ways: by a term/epigraph for each sedation-agitation level and by a specific description of the expected behavior at that level. The researchers gave 2 values for the RASS scale: one based solely on the epigraph (RASSe), which corresponds to the observer's subjective opinion, and the other according to the objective description of the patient's behavior or conduct (RASSc). As the scale is based on expected behaviors in adults (RASSc), an attempt was made to see whether differences with pediatric behavior affect the level at which a child is classified, observing if they coincide with the expert's opinion (RASSe) or not. A previously published Spanish version of the RASS was used (Supplemental Appendix 3) (26).

The COMFORT-B scale is composed of 6 items (25). Each one is scored from 1-5, obtaining a minimum score of 6 points and a maximum of 30. It distinguishes among 3 levels of sedation/agitation, for which the authors recommend associating it to a second subjective scale, such as the NISS: over-sedation (6–10 points), optimum sedation (included in the range 11–22, combined with NISS = 2), and under-sedation (23–30) (3, 5). The NISS is a 3-point scale based on the nurse expert opinion, where score 1 corresponds to insufficient sedation, 2 = adequate sedation, and 3 = oversedation (5).

The NRS is a subjective scale of 11 points which represents the expert opinion of the observer, ranging 0–10: 0 corresponds to the deepest sedation state imaginable for the patient, and 10 to the maximum agitation state.

### Analgosedation

The analgosedation protocol of the leading hospital was followed, based on prioritizing the adjustment of analgesia first sedation. Drugs and dosages were prospectively registered. The analgesics used were fentanyl, morphine, paracetamol, metamizole, ketorolac, and gabapentin. The sedatives used were propofol, dexmedetomidine, clonidine, midazolam, sevoflurane, ketamine, chlorpromazine, and levomepromazine.

### Statistical Analysis

A descriptive analysis was performed. Qualitative variables were described by absolute and relative frequencies and quantitative variables by median and interquartile range (IQR) as they did not have a normal distribution (measured by the Kolmogorov-Smirnov test). The observations which weren't assessed by the 4 observers in each group were not included in each specific analysis.

The validation stages and their statistical analysis were made following the Consensus-based Standards for the selection of health Measurement Instrument (COSMIN) criteria (23).

#### Reliability

Inter-rater reliability was measured using the intraclass correlation coefficient (ICC) two-way mixed-effects model for the COMFORT-B and NRS scales, or the quadratic weighted kappa (κ_w_) index for the RASS scale (RASSe and RASSc), among all the researchers and between the group of nurses, pediatricians, and nurses-pediatricians. Additionally for the RASS, we analyzed separately patients younger and older than 12 months. The same was done with the subgroup of restless and/or agitated patients (RASS +1 to +4), since there could be differences between anxious or agitated behavior of adults and pediatric patients. An ICC value of >0.8 and a kappa index >0.8 were considered excellent, >0.6 satisfactory or good and >0.4 moderate, according to the Landis and Koch criteria (27).

#### Construct Validity

To test construct validity, we explored the degree to which the RASS score was consistent with the following hypotheses: (1) The RASS score increases and decreases in the same direction as the COMFORT-B and the NRS do. This correlation was measured using the Spearman correlation coefficient (rho), expected to be ≥0.5. This analysis was repeated in the subgroup of children under 12 months of age. Following COSMIN criteria, rho ≥0.5 was considered as indicating that both instruments measure a similar construct, rho 0.3–0.5 as the construct is related but dissimilar, and rho < 0.3 as measuring unrelated constructs (23). (2) The RASS can distinguish between 3 different categories of sedation-agitation, similar to those of the COMFORT-B scale. We considered the ranks (−5 to −4, deep sedation), (−3 to +1, moderate and light sedation) and (+2 to +4, agitation) of the RASS to be similar to the ranks (6–10), (11–22) and (23–30) of the COMFORT-B. We used the Kruskal-Wallis test to analyze the ability of the RASS to discriminate among the 3 categories, and κ and κ_w_ indices to measure the agreement between RASS and COMFORT-B. (3) RASSe and RASSc measure the same construct (sedation-agitation). Spearman correlation coefficient was calculated, expected to be ≥0.5. (4) RASSe and RASSc scores match when rating an episode of sedation-agitation. The agreement between RASSe and RASSc was calculated using κ and κ_w_.

#### Responsiveness

A responsiveness analysis to sedative changes was carried out, rating the differences in RASS values before and after a required intervention of increase or decrease of sedatives using the Wilcoxon test for paired samples.

All analyses were performed with SPSS and STATA statistical package and a *p* value < 0.05 was considered significant.

## Results

### Patient Characteristics and Episodes

**Figure 1:**
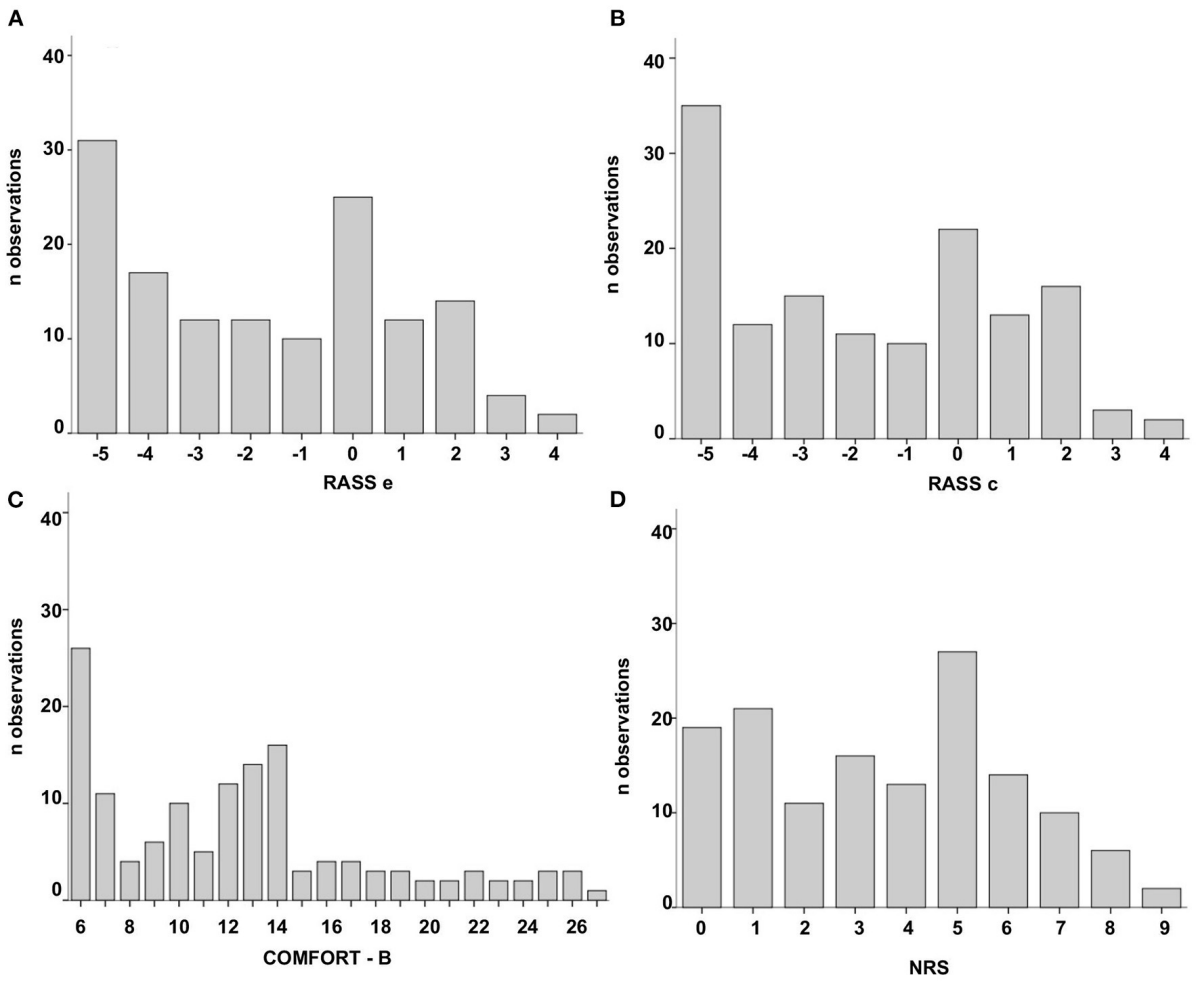
Scores of the 546 observations in 139 patient episodes according to the scale used: **(A)** Richmond Agitation-Sedation Scale based on the epigraph (RASSe). **(B)** Richmond Agitation-Sedation Scale based on the description of the conduct (RASSc). **(C)** COMFORT Behavior Scale (COMFORT-B). **(D)** Numeric Rating Scale (NRS).

Fifty-five patients (58% female) with a median age of 3.6 years (IQR: 0.7–7.8), ranging from 44 days to 16 years of age were enrolled. We obtained 146 episodes, 7 of which were excluded due to recording failures, so that 139 episodes were finally included. The characteristics are shown in Table 1. The distribution of the scores according to the different scales used is shown in Figure 1. Ten different observations were missed for every scale. There were 5 investigators who did not assess all their corresponding episodes: 1 observer missed 4 episodes, 2 observers missed 2 episodes each, and 1 observer missed 1 episode, making a total of 10 episodes in 6 patients. The patients belonged to different age groups, with 12.8, 0.6, 16.1, 1.2, 0.8, and 6.4 years, respectively.

**Table 1:** Patient characteristics and episodes.

**Category**	**Variations**	***N*** **(%)**
Number of patients		55
Age distribution	<12 months	17 (30.9%)
	12–24 months	5 (9.1%)
	2–5 years	15 (27.3%)
	6–12 years	11 (20%)
	13–19 years	7 (12.7%)
Diagnosis	Sedation for procedures	19 (34.5%)
	Respiratory Failure	10 (18.2%)
	Postoperative of cardiac surgery	9 (16.4%)
	Postoperative of otorhinolaryngological surgery	7 (12.7%)
	Postoperative of orthopedic surgery	4 (7.3%)
	Severe infections	4 (7.3%)
	Post cardiac catheterization	3 (5.4%)
	Endocrine failure	1 (1.8%)
Number of episodes		139
Number of episodes per patient	1	14 (25.5%)
	2	23 (41.8%)
	3	9 (16.4%)
	4	2 (3.6%)
	5	4 (7.3%)
	6	1 (1.8%)
	8	1 (1.8%)
	10	1 (1.8%)
Median of episodes per patient (range)		2 (1–10)
Episodes with invasive mechanical ventilation		52 (37.4%)
Episodes without sedation (%)		19 (13.7%)
Number of observations	RASS^a^	546
	COMFORT-B^b^	546
	NRS^c^	546

a *Richmond Agitation-Sedation Scale*;

b *COMFORT Behavior Scale*;

c *Numeric Rating Scale*.

The assessments carried out in each Unit are shown in the (Supplementary Table 1).

### Inter-Rater Reliability

For the RASS, 538 observations were analyzed, 528 for the COMFORT-B scale, and 532 for the NRS.

The median (IQR) RASSe and RASSc scores was −2 (−4 to 0), for both global nursing and global pediatrician groups (Supplementary Table 2).

For the COMFORT-B scale, it was obtained an ICC = 0.910 (95% CI, 0.883–0.931) among all researchers. Between nurses and pediatricians, the result was an ICC = 0.901 (95% CI, 0.876–0.92). For the NRS scale, an ICC = 0.913 (95% CI, 0.888–0.934) was obtained among all observers, and an ICC = 0.919 (95% CI, 0.898–0.935) between nurses and pediatricians (Supplementary Table 3).

RASS inter-rater reliability among all researchers and between nursing and pediatric groups is shown in Table 2. A similar result was achieved when taking a unique first observation of each patient (*n* = 55), with a κ_w_ = 0.954 (95% CI, 0.93–0.98) for RASSe, and κ_w_ = 0.961 (95% CI, 0.94–0.98) for RASSc, between nurses and pediatricians. There were no differences in inter-rater reliability between patients younger and older than 12 months [RASSe κ_w_ = 0.941 (95% CI, 0.90–0.98) vs 0.932 (95% CI, 0.914–0.949) and RASSc κ_w_ = 0.951 (95% CI, 0.917–0.985) vs. 0.944 (95% CI, 0.926–0.961)] (Supplementary Table 4).

**Table 2:** Inter-rater reliability of the Richmond Agitation-Sedation Scale (*p* < 0.001 in all cases).

**Observers group**	**κ_w_ (95 CI%)^a^**	
	**RASSe^b^**	**RASSc^c^**
Between nurses	0.927 (0.894–0.961)	0.948 (0.917 - 0.979)
Between pediatricians	0.943 (0.913–0.973)	0.942 (0.911–0.973)
Between nurses and pediatricians	0.933 (0.908–0.959)	0.946 (0.924–0.969)
Global (*n* = 538 observations)	0.934 (0.917–0.951)	0.946 (0.929–0.962)

a *Weighted kappa (95% confidence interval)*;

b *Richmond Agitation-Sedation Scale based on the epigraph*;

c *Richmond Agitation-Sedation Scale based on the description of the conduct*.

There were 27 and 28 episodes classified as restless or agitated according to RASSe and RASSc (108 and 111 observations, respectively, with RASS +1 to +4). In analyzing these observations, the inter-rater reliability of the RASS was only moderate. For RASSe, we obtained a κ_w_ = 0.527 (95% CI, 0.374–0.671), with no differences between children younger and older than 12 months [0.561 (0.637–0.755) vs. 0.478 (0.286–0.671)]. For RASSc, we observed a κ_w_ = 0.511 (95% CI, 0.345–0.678) with no differences between those younger and older than 12 months [κ_w_ = 0.509 (95% CI, 0.260–0.759) vs. 0.487 (95% CI, 0.282–0.692)].

### Construct Validity

The results of the analyses undertaken to test construct validity are shown below:

The Spearman rho correlation between the COMFORT-B scale and RASSe and RASSc was analyzed in 544 observations. The results, with rho = 0.935 (*p* < 0.001) in the global population and in the subgroups of children younger and older than 12 months, are shown in Table 3. The correlation was also statistically significant both in nursing staff (rho = 0.927 and 0.938; *p* < 0.001) and among pediatricians (rho = 0.939 and 0.931; *p* < 0.001), for RASSe and RASSc, respectively.

**Table 3:** Spearman rho correlation between RASS and COMFORT-B and between RASS and NRS, in the global population and in children < or > 12 months (*p* < 0.001 in all cases).

**Scale**	**RASS^a^**	**Global**	***N***	**≤12 months**	***n***	**>12 months**	***n***
COMFORT-B^d^	RASSe^b^	0.932	544	0.938	147	0.931	397
COMFORT-B	RASSc^c^	0.935	544	0.941	147	0.932	397
NRS^e^	RASSe	0.960	544	0.963	147	0.957	397
NRS	RASSc	0.958	544	0.967	147	0.953	397

a *Richmond Agitation-Sedation Scale*;

b *Richmond Agitation-Sedation Scale based on the epigraph*;

c *Richmond Agitation-Sedation Scale based on the description of the conduct*;

d *COMFORT Behavior Scale*;

e *Numeric Rating Scale*.

The Spearman rho correlation between RASS and NRS, in 544 observations, is shown in Table 3. It was statistically significant among nurses (rho = 0.949 and 0.948; *p* < 0.001) and pediatricians (rho = 0.973 and 0.970; *p* < 0.001), for RASSe and RASSc, respectively.

To check whether the fact that the same observer applied the 3 scales simultaneously could have facilitated the correlation between them, a randomized representative sample of procedures was analyzed ensuring that the same observer had only applied one of the scales. Similar data were obtained for all correlations (data not shown).

RASSe and RASSc scores were significantly different for the 3 sedation-agitation categories of the COMFORT-B scale (Kruskal-Wallis, *p* < 0.001) (Supplementary Table 5).

The agreement between RASS and COMFORT-B scores in classifying the patients into the 3 COMFORT-B categories is shown in Table 4, with a κ = 0.762 (95% CI, 0.713–0.811), in 544 observations. There were 26 observations that RASSe scored +2 to +4, and COMFORT-B scored 11 to 22. Among them, 88.4% (23/26) had a score of 17 or higher on the COMFORT-B scale.

**Table 4:** Agreement between the Richmond Agitation-Sedation Scale and the COMFORT behavior scale categories (*p* < 0.001 in all cases).

**Scale**	**RASS^a^**	**COMFORT-B^d^**			
	**Sedation-agitation category**	**6–10**	**1–22**	**23–30**	***n*** **observations**
RASSe^b^	−5 to −4	183	3	0	186
	−3 to +1	46	238	1	285
	+2 to +4	0	26	47	73
	*n* observations	229	267	48	544
	κ (95% CI)^e^	0.762 (0.713–0.811)			
	κ_w_ (95% CI)^f^	0.835 (0.799–0.871)			
RASS c^c^	−5 to −4	182	9	0	191
	−3 to +1	47	238	2	287
	+2 to +4	0	20	46	66
	*n* observations	229	267	48	544
	κ (95% CI)^e^	0.754 (0.703–0.804)			
	κ_w_ (95% CI)^f^	0.827 (0.789–0.865)			

***Sedation-agitation categories: Deep sedation**: RASS −5 to −4 and COMFORT-B 6 to 10. **Moderate to light sedation**: RASS−3 to +1 and COMFORT-B 11 to 22. **Agitation:** RASS +2 to +4 and COMFORT-B (23 to 30)*.

a *Richmond Agitation-Sedation Scale*;

b *Richmond Agitation-Sedation Scale based on the epigraph*;

c *Richmond Agitation-Sedation Scale based on the description of the conduct*.

d *COMFORT Behavior Scale*;

e *kappa (95% confidence interval)*;

f *weighted kappa (95% confidence interval)*.

Between RASSe and RASSc, a statistically significant correlation and agreement were observed: Spearman rho = 0.985 (*p* < 0.001), κ = 0.802 (95% CI, 0.766–0.839) and κ_w_ = 0.986 (95% CI, 0.984–0.90), in 546 observations (Supplementary Figure 1). This agreement was maintained considering the 3 sedation-agitation categories before mentioned, κ = 0.894 (95% CI, 0.859–0.928) and κ_w_ = 0.927 (95% CI, 0.902–0.951) (Supplementary Table 6).

### Responsiveness

**Figure 2:**
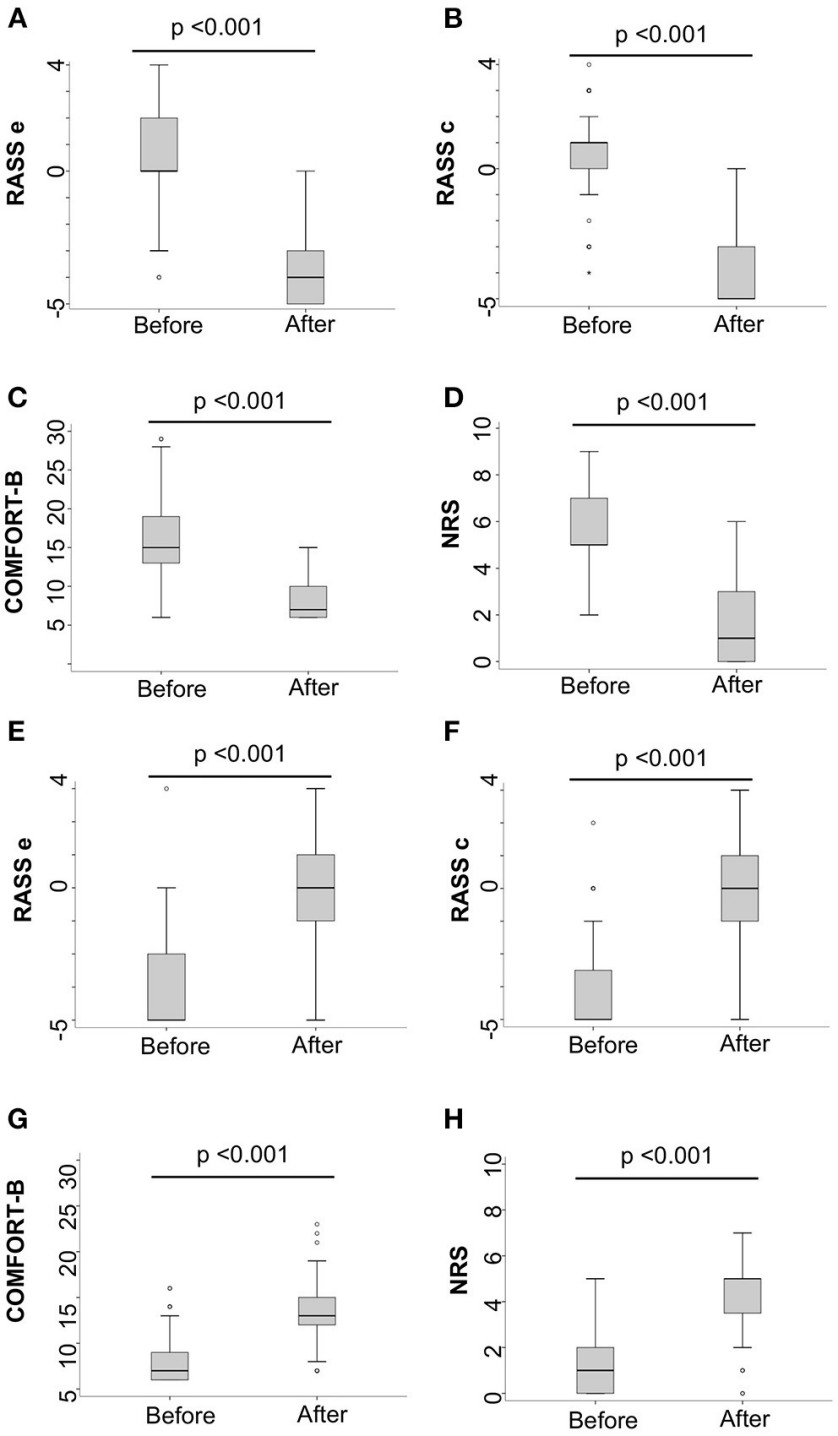
Responsiveness of the RASS: Modification in sedation-agitation scores of the different scales used, before and after a sedative intervention. **Increasing/initiation of sedatives (A)** RASSe; **(B)** RASSc; **(C)** COMFORT-B; **(D)** NRS. **Diminishing/stopping of sedatives (E)** RASSe; **(F)** RASSc; **(G)** COMFORT-B; **(H)** NRS. RASSe, Richmond Agitation-Sedation Scale based on the epigraph; RASSc, Richmond Agitation-Sedation Scale based on the description of the conduct; COMFORT-B, COMFORT Behavior Scale; NRS, Numeric Rating Scale.

To test responsiveness, 45 interventions were analyzed before and after a change in the sedative dose (18 episodes of diminishing or stopping sedation and 27 of increasing or initiation of sedation). Most of them were carried out during sedation for procedures. There was a significant modification in sedation-agitation scores following both types of intervention (Table 5; Figure 2). Doses were collected but not statistically analyzed because of the large variability in the type of drugs and dose received by each patient due to the heterogeneity of the sample and are not shown in the study.

**Table 5:** Median scores (IQR) of the 45 episodes assessed before and after an intervention of increase or decrease in sedatives.

**Scale**	**Score^e^** **Intervention**							
	**Sedative increase (*****n*** **episodes = 27)**				**Sedative decrease (*****n*** **episodes = 18)**			
	**Before**	**After**	**Difference**	***p***	**Before**	**After**	**Difference**	***p***
RASSe^a^	0 (0–2)	−4 (−5 to −3)	−4 (−5 to −3.8)	<0.001	−5 (−5 to −3)	−1 (−2 to 0)	3 (2–4)	<0.001
RASSc^b^	1 (0–1)	−5 (−5 to −3)	−5 (−6 to −3.8)	<0.001	−5 (−5 to −3)	−1 (−8 to 0)	3 (2–4.8)	<0.001
COMFORT-B^c^	15 (13–19)	7 (6–15)	−8 (−11 to −6)	<0.001	7 (6–9)	1 (12–15)	6 (3–8)	<0.001
NRS^d^	5 (5–7)	1 (0–3)	−4 (−5 to −3)	<0.001	1 (0–2)	5 (3.5–5)	4 (2–4)	<0.001

a *Richmond Agitation-Sedation Scale*;

b *Richmond Agitation-Sedation Scale based on the epigraph*;

c *Richmond Agitation-Sedation Scale based on the description of the conduct*;

d *COMFORT Behavior Scale*;

e *Numeric Rating Scale*;

*^e^Median score*.

## Discussion

Our study shows that the RASS has good measurement properties in assessing sedation in critically ill children, with and without mechanical ventilation, in terms of inter-rater reliability, construct validity and responsiveness.

It confirms some of the findings of two previous studies conducted in PICU (21, 22). Furthermore, our study has certain characteristics that strengthen our results. Firstly, a multicenter study reduces the risk of bias in the application of the results. Secondly, a video recording format ensures total independence in assessments among researchers. Third, a validated scale has been used as a comparative tool of the agitation range. And lastly, a responsiveness study has been included.

We found RASS inter-rater reliability to be excellent among health professionals from different PICUs, with similar results between pediatricians and nurses, and between patients younger and older than 12 months. These findings coincide with the two previous studies, where it was found a κ_w_ of 0.825 (*p* < 0.0001) and 0.86 (95% CI), respectively (21, 22). The inter-rater reliability, however, resulted lower in our subgroup of agitated patients.

Inter-rater reliability among the research team was also excellent in the scales chosen as references, COMFORT-B and NRS, demonstrating the ability of the collaborators to participate in the study. The researchers were experienced PICU personnel, and all had previously received a training course to use the RASS and COMFORT-B scales. This fact coincides with that observed by Kihlstrom et al. who found an improvement in the inter-rater reliability after an educational intervention for the use of the RASS in their PICU (22).

We obtained a high correlation between COMFORT-B and RASS scales, which was similar in children younger and older than 12 months. In the study by Kerson et al. a good correlation was also obtained between the RASS and the UMSS, which exclusively includes sedation levels (21). Our study is the first to validate the agitation area of the RASS with a recommended and validated tool such as the COMFORT-B scale. A high correlation with the expert's subjective opinion was observed in both studies, using a VAS in the former and a NRS in ours (21).

This study has demonstrated the RASS capacity to classify PICU patients into 3 different categories of sedation-agitation (deep sedation: RASS−5 to−4, moderate to light sedation: RASS−3 to +1, and agitation: RASS + 2 to + 4), based on the 3 levels established by the COMFORT-B scale: over-sedation (score 6–10), optimum sedation (included in the range 11–22) and under-sedation (23–30). Good agreement was observed between the 2 scales when categorizing the patients into these 3 levels. Interestingly, 9.7% of the patients who were considered as adequately sedated according to the COMFORT-B scale (scores 11–22) were assessed as agitated by the RASS. Most of these patients (88.4%) had COMFORT-B scores ≥17. This data is consistent with the pain management algorithm published by van Dijk et al. and the results obtained by Valkenburg et al., in a study conducted to validate the COMFORT-B scale to assess pain and distress (25, 28). These authors found that the cut-off point for agitation due to pain on the COMFORT-B scale was 17 and not 22 (28). Moreover, Ista et al., in a validation study of the levels of sedation of the COMFORT-B, observed that patients classified within the range of 11–22 had a 15% probability of being under-sedated, and that the correlation with the expert opinion (using the NISS) was low in this range (5). These authors conclude that the score range from 11 to 22 on the COMFORT-B scale is a “gray area,” in which “optimal sedation” would be included, but for which final interpretation is necessary to associate a second scale, to include the subjective opinion of the professional in charge of the patient (5).

The RASS has the advantage of integrating this second subscale, which we have called RASSe in this study, and that would correspond to the expert's subjective opinion. Comparing RASSe (subjective scale) with RASSc (objective description of the behavior of the patient for each level), an excellent agreement and correlation was observed.

The percentage of agitation episodes in this study (11% in RASSc and 13% in RASSe) was similar to other studies in critically ill children, which resulted in around 10% of the total (5, 13). Our results of RASS inter-rater reliability were worse in this agitation area. This may be due to an insufficient number of patients in this range, to the difficulty in assessing agitation for professionals, or to a limitation of the scale to assess agitation in children. In the present study the RASS has demonstrated its ability to distinguish whether the child is agitated or not, but this scale may not be accurate enough in designating the exact level of agitation in the pediatric population.

Finally, our study has been the first to analyze the responsiveness of the RASS in critically ill children, showing that the scale scores varied significantly after a required intervention of increase or decrease of sedatives, which makes it useful for controlling sedation modifications in PICU.

### Limitations

Our study has several limitations. The recruitment of patients was carried out according to the availability of the PI and not randomly. The sample size was not sufficient to perform an analysis by pediatric age groups. The number of patients under 9 weeks was insufficient for drawing conclusions in this age range, which is particularly important as sustained eye contact maturation is achieved at 6–9 weeks of age (29, 30). The fact that the same observer applied the 3 scales simultaneously could have facilitated the correlation between them.

### Future Research

Since the duration of eye contact, greater or <10 s, is the criterion that discriminates between RASS levels−1 and−2, it would be convenient to study infants under 9 weeks of age in more detail, assessing the need to modify the scale to adapt it to their normal psychomotor development, as Kihlstrom et al. did for neonates (22).

Regarding our results in the subgroup of distressed patients, that could be interpreted as an only moderate ability of the RASS to assess the exact degree of agitation in children future research may be needed at this range of the scale. We believe a RASS modification in the agitation area, including more typical pediatric agitation behaviors, could improve distress evaluation in critically ill children.

## Conclusions

In this multi-center study, we found a high inter-rater reliability, excellent construct validity and adequate responsiveness to change in sedative doses, of the RASS in PICU patients. The RASS also proved its ability to categorize the patients' level of sedation into deep sedation, moderate to light sedation, and agitation, enabling its use in a target-level of sedation-based protocol. This good measurement properties makes the RASS a useful instrument for monitoring sedation in PICU.

As inter-rater reliability was only moderate in the subgroup of agitated children, it may be necessary to extend future validation studies in this range.

## Data Availability Statement

The raw data supporting the conclusions of this article will be made available by the authors, without undue reservation.

## Ethics Statement

The studies involving human participants were reviewed and approved by Comité de Ética de la Investigación del Hospital Universitario Ramón y Cajal (code RASS-UCIP-01). Written informed consent to participate in this study was provided by the participants' legal guardian/next of kin.

## Collaborators of the Analgesia and Sedation Working Group of the Spanish Society of Pediatric Intensive Care (SECIP)

The following collaborators helped with the acquisition of data (scoring the videotaped clinical cases): Esther Aleo, MD, PhD, Chief of Division, Pediatric Intensive Care Unit, Hospital Universitario Clínico San Carlos, Associate Professor, Universidad Complutense de Madrid, Madrid, Spain; Elda Dolate, RN, BSN, Pediatric Nurse Specialist, Pediatric Intensive Care Unit, Hospital Universitario Niño Jesús, Madrid, Spain; Ana Estalella-Mendoza, MD, Pediatric Intensive Care Unit, Hospital Universitario Puerta del Mar, Cádiz, Spain; Isabel M Figeroa, RN, BSN, Pediatric Intensive Care Unit, Hospital Universitario Puerta del Mar, Cádiz, Spain; José Fernández-Cantalejo, MD, PhD, Pediatric Intensive Care Unit, Hospital Universitario Fundación Jiménez Díaz, Madrid, Spain; Francisco Fernández-Carrión, MD, Pediatric Intensive Care Unit, Hospital Universitario de Salamanca, Spain; Sonia Fuentes, RN, BSN, Pediatric Intensive Care Unit, Hospital Regional Universitario de Málaga, Spain; Marta García, RN, BSN, Pediatric Nurse Specialist, Pediatric Intensive Care Unit, Hospital Universitario Ramón y Cajal, Madrid, Spain; Miguela García-Cervigon, RN, BSN, Pediatric Intensive Care Unit, Hospital General La Mancha Centro, Alcázar de San Juan, Spain; Federico Goded, MD, Pediatric Intensive Care Unit, Hospital Universitario La Paz, Madrid, Spain; M del Pilar Hernández, RN, BSN, Pediatric Intensive Care Unit, Hospital Universitario de Salamanca, Spain; María Hernández, RN, BSN, Pediatric Intensive Care Unit, Hospital Universitario Doce de Octubre, Madrid, Spain; David Lozano, MD, PhD, Pediatric Intensive Care Unit, Hospital General La Mancha Centro, Alcázar de San Juan, Spain; M Luisa Luaces, RN, BSN, Pediatric Nurse Specialist, Pediatric Intensive Care Unit, Hospital Universitario Ramón y Cajal, Madrid, Spain; Virginia Manzano, RN, BSN, Pediatric Nurse Specialist, Pediatric Intensive Care Unit, Hospital Universitario Ramón y Cajal, Madrid, Spain; Ana Marcos, MD, Pediatric Intensive Care Unit, Hospital Universitario Virgen de la Arrixaca, Murcia, Spain; Blanca Mayordomo, RN, BSN, Pediatric Nurse Specialist, Pediatric Intensive Care Unit, Hospital Clínico Universitario San Carlos, Madrid, Spain; Elena Montero, RN, BSN, Pediatric Intensive Care Unit, Hospital Universitario Fundación Jiménez Díaz, Madrid, Spain; Raúl Montero-Yéboles MD, Pediatric Intensive Care Unit, Hospital Universitario Reina Sofía, Córdoba, Spain; Alba Palacios, MD, Pediatric Intensive Care Unit, Hospital Universitario Doce de Octubre, Madrid, Spain; Patricia Paredes, RN, BSN, Pediatric Intensive Care Unit, Hospital General Universitario Gregorio Marañón, Madrid, Spain; Francisco Piedras, RN, BSN, Pediatric Intensive Care Unit, Hospital Universitario Reina Sofía, Córdoba, Spain; This collaborator helped with acquisition of data (scoring the videotaped clinical cases); Mónica Riaza, MD, Pediatric Intensive Care Unit, Hospital Universitario Montepríncipe, Madrid, Spain; Daniel Sánchez, intensive care ARNP, BSN, Pediatric Intensive Care Unit, Hospital Universitario Niño Jesús, Madrid, Spain; Josefa Sosa, RN, BSN, Pediatric Nurse Specialist, Pediatric Intensive Care Unit, Hospital Universitario Ramón y Cajal, Madrid, Spain; José Luis Unzueta, MD, Pediatric Intensive Care Unit, Hospital Universitario Niño Jesús, Madrid, Spain; Cristina Yun, MD, Department of Pediatrics, Pediatric Intensive Care Unit, Hospital Regional Universitario de Málaga, Spain.

## Author Contributions

RT: this author helped with conception, design, acquisition, analysis, and interpretation of data for the work, drafting the work, giving the final approval of the version to be published, and agrees to be accountable for all aspects of the work. JL-H: this author helped with the interpretation of data for the work, drafting and revising the work critically for important intellectual content, giving the final approval of the version to be published, and agrees to be accountable for all aspects of the work. ÁA: this author helped with the statistical analysis and interpretation of data for the work, drafting the work, giving the final approval of the version to be published, and agrees to be accountable for all aspects of the work. JC: this author helped with interpretation of data, English language editing, giving the final approval of the version to be published, and agrees to be accountable for all aspects of the work SM: this author helped with acquisition and interpretation of data for the work, drafting and revising the work critically for important intellectual content, giving the final approval of the version to be published, and agrees to be accountable for all aspects of the work. All authors contributed to the article and approved the submitted version.

## Funding

This study received financial support from the IRYCIS (Instituto Ramón y Cajal de Investigación Sanitaria).

## Conflict of Interest

The authors declare that the research was conducted in the absence of any commercial or financial relationships that could be construed as a potential conflict of interest.

## Publisher's Note

All claims expressed in this article are solely those of the authors and do not necessarily represent those of their affiliated organizations, or those of the publisher, the editors and the reviewers. Any product that may be evaluated in this article, or claim that may be made by its manufacturer, is not guaranteed or endorsed by the publisher.

## Nomenclature

CI, Confidence interval; COMFORT-B scale, COMFORT behavior scale; COSMIN: Consensus-based Standards for the selection of health Measurement Instruments; ICC, Intraclass correlation coefficient; IQR, Interquartile range; κ, Kappa; κ_w_, Weighted kappa; NISS, Nurse Interpretation of Sedation Score; NRS, Numeric rating scale; pCAM-ICU, Pediatric Confusion Assessment Method for the ICU; psCAM-ICU, Preschool Confusion Assessment Method for the ICU; PICU, Pediatric intensive care unit; RASS, Richmond Agitation-Sedation Scale; RASSe, RASS by epigraph; RASSc, RASS by description of the patient conduct/behavior; rho, Spearman correlation coefficient; SECIP, Spanish Society of Pediatric Intensive Care; UMSS, University of Michigan Sedation Scale; VAS, visual analogue sedation-agitation scale.

## References

[1] Harris J Ramelet AS van Dijk M Pokorna P Wielenga J Tume L. Clinical recommendations for pain, sedation, withdrawal and delirium assessment in critically ill infants and children: an ESPNIC position statement for healthcare professionals. Intensive Care Med. (2016) 42:972–86. 10.1007/s00134-016-4344-1 27084344 PMC4846705

[2] Lucas SS Nasr VG Ng AJ Joe C Bond M DiNardo JA. Pediatric cardiac intensive care society 2014 consensus statement: pharmacotherapies in cardiac critical care: sedation, analgesia and muscle relaxant. Pediatr Crit Care Med. (2016) 17(3 Suppl 1):S3–15. 10.1097/PCC.0000000000000619 26945327

[3] Vet NJ Kleiber N Ista E de Hoog M de Wildt SN. Sedation in critically ill children with respiratory failure. Front Pediatr. (2016) 4:89. 10.3389/fped.2016.00089 27606309 PMC4995367

[4] De Jonghe B Cook D Appere-De-Vecchi C Guyatt G Meade M Outin H. Using and understanding sedation scoring systems: a systematic review. Intensive Care Med. (2000) 26:275–85. 10.1007/s001340051150 10823383

[5] Ista E van Dijk M Tibboel D de Hoog M. Assessment of sedation levels in pediatric intensive care patients can be improved by using the COMFORT ”behavior" scale. Pediatr Crit Care Med. (2005) 6:58–63. 10.1097/01.PCC.0000149318.40279.1A 15636661

[6] Vet NJ Ista E de Wildt SN van Dijk M Tibboel D de Hoog M. Optimal sedation in pediatric intensive care patients: a systematic review. Intensive Care Med. (2013) 39:1524–34. 10.1007/s00134-013-2971-3 23778830

[7] Poh YN Poh PF Buang SN Lee JH. Sedation guidelines, protocols, and algorithms in PICUs: a systematic review. Pediatr Crit Care Med. (2014) 15:885–92. 10.1097/PCC.0000000000000255 25230314

[8] Garcia Guerra G Joffe AR Cave D Duff J Duncan S Sheppard C. Survey of sedation and analgesia practice among canadian pediatric critical care physicians. Pediatr Crit Care Med. (2016) 17:823–30. 10.1097/PCC.0000000000000864 27467012

[9] Zeilmaker-Roest GA Wildschut ED van Dijk M Anderson BJ Breatnach C Bogers AJJC. An international survey of management of pain and sedation after paediatric cardiac surgery. BMJ Paediatr Open. (2017) 1:e000046. 10.1136/bmjpo-2017-000046 29637103 PMC5862168

[10] van Dijk M de Boer JB Koot HM Tibboel D Passchier J Duivenvoorden HJ. The reliability and validity of the COMFORT scale as a postoperative pain instrument in 0 to 3-year-old infants. Pain. (2000) 84:367–77. 10.1016/S0304-3959(99)00239-0 10666543

[11] Maaskant J Raymakers-Janssen P Veldhoen E Ista E Lucas C Vermeulen H. The clinimetric properties of the COMFORT scale: a systematic review. Eur J Pain. (2016) 20:1587–611. 10.1002/ejp.880 27161119

[12] Dorfman TL Sumamo Schellenberg E Rempel GR Scott SD Hartling L. An evaluation of instruments for scoring physiological and behavioral cues of pain, non-pain related distress, and adequacy of analgesia and sedation in pediatric mechanically ventilated patients: a systematic review. Int J Nurs Stud. (2014) 51:654–76. 10.1016/j.ijnurstu.2013.07.009 23987802

[13] Boerlage AA Ista E Duivenvoorden HJ de Wildt SN Tibboel D van Dijk M. The COMFORT behaviour scale detects clinically meaningful effects of analgesic and sedative treatment. Eur J Pain. (2015) 19:473–9. 10.1002/ejp.569 25070754

[14] Boerlage AA Ista E de Jong M Tibboel D van Dijk M. The COMFORT behavior scale: is a shorter observation period feasible? Pediatr Crit Care Med. (2012) 13:e124–5. 10.1097/PCC.0b013e3182192d92 21499179

[15] Curley MA Harris SK Fraser KA Johnson RA Arnold JH. State behavioral scale: a sedation assessment instrument for infants and young children supported on mechanical ventilation. Pediatr Crit Care Med. (2006) 7:107–14. 10.1097/01.PCC.0000200955.40962.38 16446601 PMC1626525

[16] Barr J Fraser GL Puntillo K Ely EW Gélinas C Dasta JF. American College of Critical Care Medicine. Clinical practice guidelines for the management of pain, agitation, and delirium in adult patients in the intensive care unit. Crit Care Med. (2013) 41:263–306. 10.1097/CCM.0b013e3182783b72 23269131

[17] Devlin JW Skrobik Y Gélinas C Needham DM Slooter AJC Pandharipande PP. Clinical practice guidelines for the prevention and management of pain, agitation/sedation, delirium, immobility, and sleep disruption in adult patients in the ICU. Crit Care Med. (2018) 46:e825–73. 10.1097/CCM.0000000000003299 30113379

[18] Smith HA Boyd J Fuchs DC Melvin K Berry P Shintani A. Diagnosing delirium in critically ill children: validity and reliability of the pediatric confusion assessment method for the intensive care unit. Crit Care Med. (2011) 39:150–7. 10.1097/CCM.0b013e3181feb489 20959783 PMC3776416

[19] Smith HA Gangopadhyay M Goben CM Jacobowski NL Chestnut MH Savage S. The preschool confusion assessment method for the icu: valid and reliable delirium monitoring for critically ill infants and children. Crit Care Med. (2016) 44:592–600. 10.1097/CCM.0000000000001428 26565631 PMC4764386

[20] Traube C Silver G Kearney J Patel A Atkinson TM Yoon MJ. Cornell assessment of pediatric delirium: a valid, rapid, observational tool for screening delirium in the PICU^*^. Crit Care Med. (2014) 42:656–63. 10.1097/CCM.0b013e3182a66b76 24145848 PMC5527829

[21] Kerson AG DeMaria R Mauer E Joyce C Gerber LM Greenwald BM. Validity of the richmond agitation-sedation scale (RASS) in critically ill children. J Intensive Care. (2016) 4:65. 10.1186/s40560-016-0189-5 27800163 PMC5080705

[22] Kihlstrom MJ Edge AP Cherry KM Zarick PJ Beck SD Boyd JM. Multi-modal Educational Curriculum To Improve Richmond Agitation-Sedation Scale Inter-Rater Reliability In Pediatric Patients. Pediatr Qual Saf. (2018) 3:e096. 10.1097/pq9.0000000000000096 30584623 PMC6221595

[23] Mokkink LB Prinsen CA Terwee CB de Vet H Bouter L Patrick D. Consensus-Based Standards for the Selection of Health Measurement Instruments-COSMIN. Amsterdam: COSMIN (2015). 10.1590/bjpt-rbf.2014.0143 PMC4900032 26786084

[24] Sessler CN Gosnell MS Grap MJ Brophy GM O'Neal PV Keane KA. The richmond agitation-sedation scale: validity and reliability in adult intensive care unit patients. Am J Respir Crit Care Med. (2002) 166:1338–44. 10.1164/rccm.2107138 12421743

[25] van Dijk M Peters JW van Deventer P Tibboel D. The COMFORT behavior scale: a tool for assessing pain and sedation in infants. Am J Nurs. (2005) 105:33–6. 10.1097/00000446-200501000-00019 15659992

[26] Rojas-Gambasica JA Valencia-Moreno A Nieto-Estrada VH Méndez-Osorio P Molano-Franco D Jiménez-Quimbaya AT. Transcultural and linguistic adaptation of the richmond agitation-sedation scale to spanish. Colomb J Anesthesiol. (2016) 44:216–21 10.1016/j.rcae.2016.04.005

[27] Landis JR Koch GG. The measurement of observer agreement for categorical data. Biometrics. (1977) 33:159–74. 10.2307/2529310 843571

[28] Valkenburg AJ Boerlage AA Ista E Duivenvoorden HJ Tibboel D van Dijk M. The COMFORT-behavior scale is useful to assess pain and distress in 0- to 3-year-old children with down syndrome. Pain. (2011) 152:2059–64. 10.1016/j.pain.2011.05.001 21640484

[29] García-Alix A Quero J. Pares craneales relacionados con sentidos especiales. El primero, el segundo y el octavo par craneal. In: García-Alix A Quero J editors. Evaluación Neurológica Del Recién Nacido. Madrid: Díaz de Santos (2010). 397–452 pp.

[30] Moller HU Larsen DA. Milestones and normative data. In: Lambert SR Lyons CJ editors. Taylor and Hoyt's Pediatric Ophthalmology and Strabismus. 5th ed. Edinburgh: Elsevier (2016). 40–9 pp.

