# Supplementary material for: Validity and Reliability of the Richmond Agitation-Sedation Scale in Pediatric Intensive Care Patients: A Multicenter Study
Source: Front Pediatr. 2022 Jan 3;9:795487. doi: 10.3389/fped.2021.795487 (PMC8762108; doi:10.3389/fped.2021.795487)
Supplement: Supplementary file 1 [file Data_Sheet_1.docx]

Supplementary Material

# Supplementary Appendix

## Supplementary Appendix 1. Richmond Agitation-Sedation Scale

| **Supplemental Appendix 1.** Richmond Agitation-Sedation Scale (RASS) | | |
| --- | --- | --- |
| Score | Term | Description |
| +4 | Combative | Overtly combative or violent; immediate danger to staff |
| +3 | Very agitated | Pulls on or removes tube(s) or has aggressive behavior towards staff |
| +2 | Agitated | Frequent non purposeful movement or patient-ventilator dyssynchrony |
| +1 | Restless | Anxious or apprehensive but movements not aggressive or vigorous |
| 0 | Alert and calm |  |
| -1 | Drowsy | Not fully alert, but has sustained (more than 10 seconds) awakening, with eye contact, to voice |
| -2 | Light sedation | Briefly (less than 10 seconds) awakens with eye contact to voice |
| -3 | Moderate sedation | Any movement (but no eye contact) to voice |
| -4 | Deep sedation | No response to voice, but any movement to physical stimulation |
| -5 | Unarousable | No response to voice or physical stimulation |
| From Sessler et al.^24^ The procedure described by the authors is not shown.^24^ | | |

## Supplementary Appendix 2. COMFORT Behavior Scale

| **Supplemental Appendix 2.** COMFORT Behavior Scale | | |
| --- | --- | --- |
| Alertness | Deeply asleep (eyes closed, no response to changes in the environment) | 1 |
|  | Lightly asleep (eyes mostly closed, occasional responses) | 2 |
|  | Drowsy (child closes his or he eyes frequently, less responsive to the environment) | 3 |
|  | Awake and alert (child responsive to the environment) | 4 |
|  | Awake and hyperalert (exaggerated responses to environmental stimuli) | 5 |
| Calmness-Agitation | Calm (child appears serene and tranquil) | 1 |
|  | Slightly anxious (child shows slight anxiety) | 2 |
|  | Anxious (child appears agitated but remains in control) | 3 |
|  | Very anxious (child appears very agitated, just able to control) | 4 |
|  | Panicky (child appears severely distressed, with loss of control) | 5 |
| Respiratory response  (score only in mechanically ventilated children) | No spontaneous respiration | 1 |
|  | Spontaneous and ventilator respiration | 2 |
|  | Restlessness or resistance to ventilator | 3 |
|  | Active breathing against ventilator or regular coughing | 4 |
|  | Fighting against ventilator | 5 |
| Crying  (score only in children breathing spontaneously) | Quiet breathing, no crying sounds | 1 |
|  | Occasional sobbing or moaning | 2 |
|  | Whining (monotone) | 3 |
|  | Crying | 4 |
|  | Screaming or shrieking | 5 |
| Physical movement | No movement | 1 |
|  | Occasional slight movements (≤ 3) | 2 |
|  | Frequent slight movements (≥ 3) | 3 |
|  | Vigorous movements limited to extremities | 4 |
|  | Vigorous movements including torso and head | 5 |
| Muscle tone | Muscles totally relaxed, no muscle tone | 1 |
|  | Reduced muscle tone, less resistance than normal | 2 |
|  | Normal muscle tone | 3 |
|  | Increased muscle tone and flexion of fingers and toes | 4 |
|  | Extreme muscle rigidity and flexion of fingers and toes | 5 |
| Facial tension | Facial muscle totally relaxed | 1 |
|  | Normal facial tone | 2 |
|  | Tension evident in some facial muscles (not sustained) | 3 |
|  | Tension evident throughout facial muscles (sustained) | 4 |
|  | Facial muscles contorted and grimacing | 5 |
| From van Dijk et al.^10,25^ The procedure described by the authors is not shown.^25^ | | |

## Supplementary Appendix 3. Richmond Agitation-Sedation Scale, Spanish version

| **Supplemental Appendix 1.** Escala de agitación y sedación de Richmond (RASS) en español | | |
| --- | --- | --- |
| Puntuación | Término | Descripción |
| +4 | Combativo | Abiertamente combativo o violento. Peligro inmediato para el personal |
| +3 | Muy agitado | Se retira tubo(s) o catéter(es) o tiene un comportamiento agresivo hacia el personal |
| +2 | Agitado | Movimiento frecuente no intencionado o asincronía paciente-ventilador |
| +1 | Inquieto | Ansioso o temeroso pero sin movimientos agresivos o vigorosos |
| 0 | Alerta and calmado |  |
| -1 | Somnoliento | No completamente alerta, pero se ha mantenido despierto(más de 10 segundos) con contacto visual, a la voz(llamado) |
| -2 | Sedación ligera | Brevemente, despierta con contacto visual (menos de 10segundos) al llamado |
| -3 | Sedación moderada | Algún movimiento (pero sin contacto visual) al llamado |
| -4 | Sedación profunda | No hay respuesta a la voz, pero a la estimulación física hay algún movimiento |
| -5 | No despierta | Ninguna respuesta a la voz o a la estimulación física |
| 1. Observe al paciente: ¿El paciente está alerta y calmado? = puntuación 0.  ¿El paciente tiene un comportamiento que sugiere inquietud o agitación? (puntuación de +1 a +4 según los criterios antes mencionados, bajo la descripción).  2. Si el paciente no está alerta, en voz alta llame al paciente por el nombre y pídale que abra los ojos y lo observe. Repítalo una vez si es necesario. Puede solicitarle al paciente que continúe observándolo. El paciente tiene apertura de ojos y contacto visual, la cual se mantiene durante más de 10 segundos (puntuación de -1). El paciente tiene apertura de ojos y contacto visual, pero esto no se mantiene durante 10 segundos (puntuación de -2). El paciente tiene cualquier movimiento en respuesta a la voz, excluyendo el contacto visual (puntuación de -3).  3. Si el paciente no presenta respuesta a la voz, estimular físicamente al paciente por medio de la agitación del hombro y luego frotando su esternón si no hay respuesta a la agitación del hombro. El paciente tiene cualquier movimiento a la estimulación física (puntuación -4).El paciente no presenta respuesta alguna a la voz o la estimulación física (puntuación -5).  Fuente: autores (Sessler et al^24^) | | |
| From Rojas-Gambasica et al.^26^ | | |

# Supplementary Tables

## Supplementary Table 1. Assessments carried out in each Pediatric Intensive Care Unit

| Supplementary Table 1. Assessments carried out in each Pediatric Intensive Care Unit | | | |
| --- | --- | --- | --- |
| **Unit** | **n collaborators** | **n assessed episodes** | **n assessments** |
| - H.U. Ramón y Cajal, Madrid | - 4 | - 78 | - 312 |
| - H.U. Niño Jesús, Madrid | - 3 | - 56 | - 224 |
| - H.U. de Salamanca | - 2 | - 46 | - 184 |
| - H.U. Reina Sofía, Córdoba | - 2 | - 41 | - 164 |
| - H.U. Clínico San Carlos, Madrid | - 2 | - 40 | - 160 |
| - H.U. Fundación Jiménez Díaz, Madrid | - 2 | - 40 | - 160 |
| - H.U. Puerta del Mar, Cádiz | - 2 | - 40 | - 160 |
| - H.U. Doce de Octubre, Madrid | - 2 | - 39 | - 156 |
| - H.U. Gregorio Marañón, Madrid | - 2 | - 38 | - 152 |
| - H.Regional U. de Málaga | - 2 | - 36 | - 144 |
| - H.General U. La Mancha Centro | - 2 | - 32 | - 128 |
| - H.U. La Paz, Madrid | - 1 | - 20 | - 80 |
| - H.U. Montepríncipe, Madrid | - 1 | - 20 | - 80 |
| - H.U. Virgen de la Arrixaca, Murcia | - 1 | - 20 | - 80 |
| - **Total** | - 28 | - 546 | - 2184 |

## Supplementary Table 2. Median scores (IQR) of the 139 episodes assessed in the different scales used according to each group of observers

| **Supplementary Table 2.**  Median scores (IQR) of the 139 episodes assessed in the different scales used according to each group of observers | | | | |
| --- | --- | --- | --- | --- |
| **Observer group** | **RASS e**^a^ | **RASS c**^b^ | **COMFORT B**^c^ | **NRS^d^** |
| Nurse 1 | - 2 (-4 to 0) | -2 (-4 to1) | 12 (7-16) | 4 (1–5.5) |
| Nurse 2 | -2 (-4 to 0) | -2 (-4 to 0) | 11 (7-15) | 3 (1-5) |
| **Nurse global** | **- 2 (-4 to 0)** | **-2 (-4 to 0)** | **12 (7-16)** | **4 (1-5)** |
| Pediatric 1 | -2 (-4 to 0) | -2 (-4 to 0) | 12 (7-14) | 4 (1-5) |
| Pediatric 2 | -2 (-4 to 0) | -2 (-4 to 0) | 12 (7-15) | 4 (1-5.5) |
| **Pediatric global** | **-2 (-4 to 0)** | **-2 (-4 to 0)** | **12 (7-15)** | **4 (1-5)** |
| ^a^RASS by epigraph; ^b^RASS by description of the conduct; ^c^COMFORT Behavior Scale; ^d^Numeric Rating Scale | | | | |

## Supplementary Table 3. Inter-rater reliability of the COMFORT-B and NRS scales, among all observers and between nurses and pediatricians [p<0.001 in all cases]

| **Supplementary Table 3.** Inter-rater reliability of the COMFORT-B and NRS scales, among all observers and between nurses and pediatricians [p<0.001 in all cases] | | | |
| --- | --- | --- | --- |
| **Scales** | **All observers** | **Nurses / Pediatricians** | **n** |
|  | **ICC (95 CI%)**^c^ | **ICC (95 CI%)**^c^ |  |
| COMFORT B^a^ | 0.910 (0.883 – 0.931) | 0.901 (0.876 – 0.921) | 528 |
| NRS^b^ | 0.913 (0.888 – 0.934) | 0.919 (0.898 – 0.935) | 532 |
| ^a^COMFORT Behavior Scale; ^b^Numeric Rating Scale; ^c^Intraclass correlation coefficient (95% confidence interval); | | | |

## Supplementary Table 4. Inter-rater reliability (κ_w_) for RASSe and RASSc in children less than and older than 12 months [p<0.001 in all cases]

| **Supplementary Table 4.** Inter-rater reliability of RASSe and RASSc in children less than and older than 12 months [p<0.001 in all cases] | | |
| --- | --- | --- |
|  | κ_w_ (95% CI)^a^ | |
|  | **RASSe**^b^ | |
|  | **<12 months** | **>12 months** |
| Between nurses | 0.917 (0.821 – 1) | 0.932 (0.906 – 0.957) |
| Between pediatricians | 0.971 (0.951 – 0.992) | 0.931 (0.889 – 0.973) |
| Between nurses and pediatricians | 0.936 (0.871 -1) | 0.932 (0.908 – 0.956) |
| **Global** (n observations=538) | **0.941 (0.90 – 0.98)** | **0.932 (0.914 – 0.949)** |
|  | **RASSc**^c^ | |
|  | **<12 months** | **>12 months** |
| Between nurses | 0.929 (0.835 – 1) | 0.959 (0.937 – 0.980) |
| Between pediatricians | 0.971 (0.950 – 0.992) | 0.914 (0.864 – 0.964) |
| Between nurses and pediatricians | 0.951 (0.902 – 0.999) | 0.945 (0.920 – 0.970) |
| **Global** (n observations=538) | **0.951 (0.917 – 0.985)** | **0.944 (0.926 – 0.961)** |
| ^a^weighted kappa (95% confidence interval);*^b^*RASS by epigraph; *^c^*RASS by description of the conduct. | | |

## Supplementary Table 5. Median RASS (IQR) scores at the 3 categories of the COMFORT-B scale.

| **Supplementary Table 5.** Median RASS (IQR)^a^ scores at the 3 categories of the COMFORT-B scale | | | | |
| --- | --- | --- | --- | --- |
|  | **COMFORT-B**^b^ | | | |
|  | **6-10** | **11-22** | **23-30** | **p*** |
| **RASSe**^c^ | -4 (-5 to -4) | 0 (-1 to +1) | 3 (2-3) | **<0.001** |
| **RASSc**^d^ | -5 (-5 to -4) | 0 (-1 to +1) | 3 (2-3) | **<0.001** |
| *Kruskal-Wallis test. ^a^Interquartile range. ^b^COMFORT-B levels of sedation, 6-10: oversedation; 11-22: optimal sedation; 23-30: undersedation. ^c^RASS by epigraph; ^d^RASS by description of the conduct. | | | | |

## Supplementary Table 6. Agreement between RASSe and RASSc according to 3 different sedation-agitation categories

| **Supplementary Table 6.** Agreement between RASSe^a^ and RASSc^b^ according to 3 different sedation-agitation categories | | | | |
| --- | --- | --- | --- | --- |
|  |  | **RASS c** | | |
| **RASS e** |  | **-5 to -4** | **-3 to +1** | **+2 to +4** |
|  | **-5 to -4** | 178 | 8 | 0 |
|  | **-3 to +1** | 13 | 269 | 3 |
|  | **+2 to +4** | 0 | 10 | 65 |
| **κ (CI95%)**^c^ | | 0.894 (0.859 - 0.928)^*^ | | |
| **κ_w_ (CI95%)**^d^ | | 0.927 (0.902-0.951)^*^ | | |
| **Sedation-agitation categories: Deep sedation:** RASS -5 to -4; M**oderate to light sedation:** -3 to +1; **Agitation:** +2 to +4. (^*^p<0.001). Total number of observations = 546.  ^a^RASS by epigraph; ^b^RASS by description of the conduct; ^c^kappa (95% confidence interval); ^d^weighted kappa (95% confidence interval). | | | | |

## Supplementary Figures

3.1 **Supplementary Figure 1**. Spearman rho correlation between RASSe and RASSc scores in 546 observations (p<0.001)


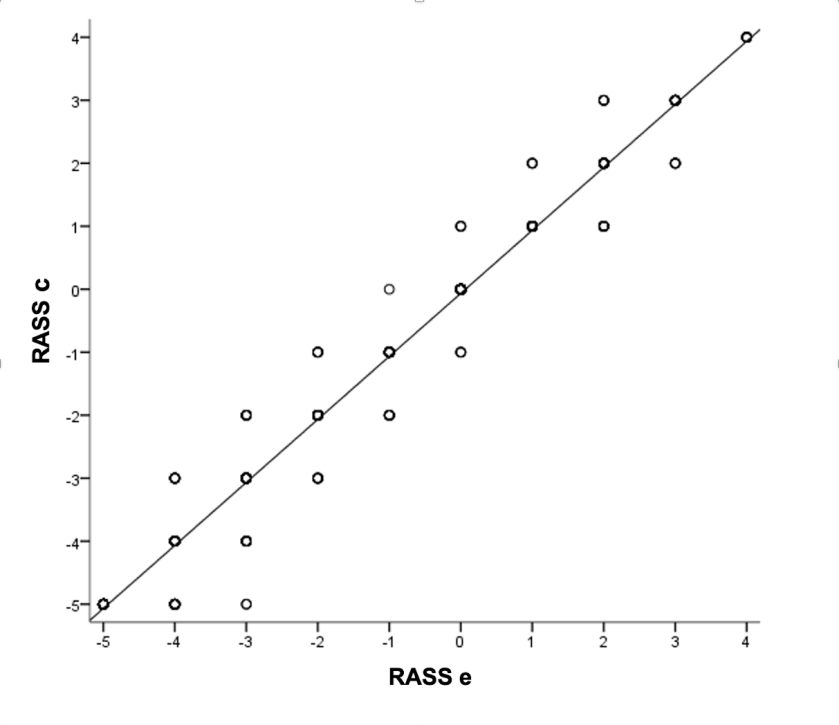


**
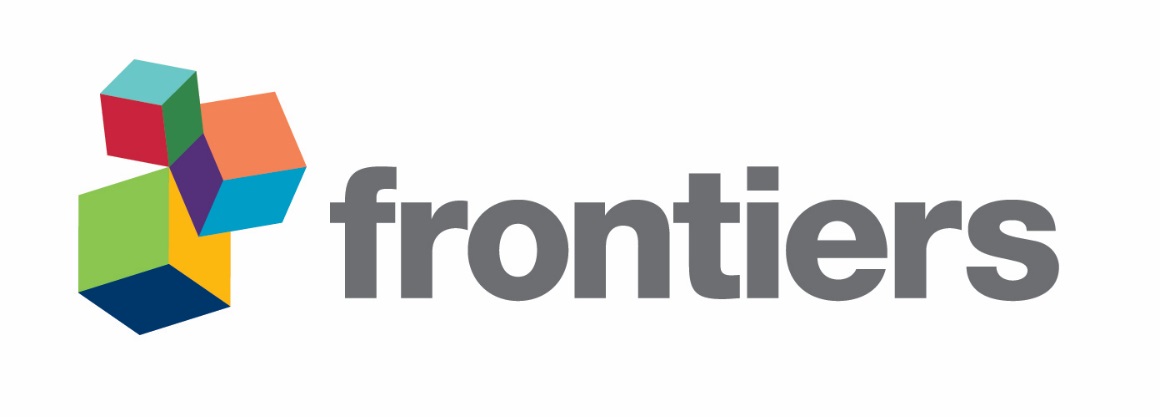
**
